# Supplementary material for: Species-Specific Response of Corals to Imbalanced Ratios of Inorganic Nutrients
Source: Int J Mol Sci. 2023 Feb 4;24(4):3119. doi: 10.3390/ijms24043119 (PMC9962417; doi:10.3390/ijms24043119)
Supplement: Supplementary file 1 [file ijms-24-03119-s001.zip › ijms-2086390-supplementary.pdf]

Species-Specific Response of Corals to Imbalanced Ratios of Inorganic Nutrients

Alice C. A. Blanckaert [1,2], Tom Biscéré [1], Renaud Grover [1] and Christine Ferrier-Pagès [1]

[1] Coral Ecophysiology Team, Centre Scientifique de Monaco, 8 Quai Antoine 1er, MC-98000 Monaco, Monaco
[2] IFD-ED 129, Sorbonne Université Sciences (Formerly UPMC Université Paris VI), CEDEX 05, 75005 Paris, France

Supplementary material

I Introduction

Table S1: Summary of the impact of different nutrient enrichment (nitrate, ammonium, phosphorus alone or in combination) on different physiological parameters of corals.
Table was based on the results presented by 47 studies. n represents the number of studies that measured this parameter. Nutrient impacts were evaluated as positive (1, green), negative (-1, red) or neutral (0, white), when diverging results were found in the literature an average were calculated.
The purpose of this table was only to have a quick overview of the parameters studied in the literature and the overall impact of nutrient, it is only meant as an informative table.

	Coral growth	Symbiont density	Chlorophyll content	Photosynthetic rates	Tissue parameters	Carbon content	Nitrogen content	Phosphorus content	Reproduction	Survival	Coral Health
**NO_3**	-0.38 (n=8)	0 (n=6)	0.29 (n=7)	-0.17 (n=12)	0 (n=3)	0 (n=1)	1 (n=1)			-0.25 (n=4)	-0.83 (n=6)
**NH_4**	-0.3 (n=10)	0.5 (n=8)	1 (n=6)	0.5 (n=4)	0.4 (n=5)	0 (n=1)	0.5 (n=2)	0 (n=1)	-1 (n=2)		0 (n=2)
**PO_4**	-0.45 (n=11)	0.4 (n=5)	-0.25 (n=4)	0.67 (n=3)	0.67 (n=3)	0.25 (n=4)	0.33 (n=3)	0 (n=2)			
**NO_3 / PO_4**	0 (n=6)	0 (n=1)	0.5 (n=4)	0.33 (n=3)	0 (n=1)	0 (n=1)	1 (n=1)				
**NH_4 / PO_4**	-0.3 (n=6)	0.4 (n=5)	0.5 (n=2)	0 (n=2)	1 (n=1)	0 (n=1)	0.5 (n=2)	0 (n=2)			
**NO_3 / NH_4**	0.5 (n=2)	-1 (n=1)	0 (n=1)	0 (n=2)	0 (n=1)	-1 (n=1)					
**NO_3 / NH_4 / PO_4**	0.25 (n=4)	1 (n=1)	1 (n=1)						-1 (n=1)		-1 (n=2)

Table S2: Summary of the carbon, nitrogen and phosphorus content and carbon-to-nitrogen, carbon-to-phosphorus and nitrogen-to-phosphorus ratios under different nutrient enrichment condition.

**Nutrient enrichment**	**Partner**	**Study**	**Context**	**Coral species**	**Concentration**	**Carbon content ($\mu g\ cm^{-2}$)**	**Nitrogen content ($\mu g\ cm^{-2}$)**	**Phosphorus content ($\mu g\ cm^{-2}$)**	**C:N** (atomic)	**C:P** (atomic)	**N:P** (atomic)
Control	Coral holobiont	Meyer & Shultz 1985	In situ (fish excretion)	*Acropora palmata*			208 ± 30	31 ± 4			21.8 ± 0.7
				Porites furcata			451 ± 0.12	44 ± 2			26.3 ± 0.7
		Tanaka et al 2010	Exp	*Montipora digitata*		3432 ± 540	392 ± 42	26.4 ± 2.5	10.2	336	33
		Cox and Ward 2002	Exp	*Montipora capitata*		40.8 ± 10.9 (%)	4.1 ± 0.23 (%)				
		Stambler et al 1991	Exp	*Stylophora pistillata*					6.1	233	38
	Animal tissue	Muscatine et al. 1989	Exp	*Stylophora pistillata*		704.7 ± 52.8	112.7 ± 5.9		7.31 ± 0.16		
		Tanaka et al 2007	Exp	*Stylophora pistillata*		4116 ± 348	644 ± 56		7.4 ± 0.1		
		Godinot et al. 2011	Exp	*Stylophora pistillata*		824.4 ± 34.8	145.6 ± 9.8	4.9 ± 0.7	6.6	432.1	65.4
		McCauley & Goulet 2019	Exp	*Pseudopleaxura porosa*					6.8		
				Eunicea tourneforti					4		
		Tanaka 2017	Exp	*Montipora digitata*		396			7.9		
				Porites cylindrica		600			6.9		
	Symbiont	Muscatine et al. 1989	Exp	*Stylophora pistillata*		204 ± 46.8	29.9 ± 6.4		7.7 ± 0.7		
		Snidvong & Kinzie 1994	Exp	*Pocillopora damicornis*		307 (pg.cell^{-1})	33 (pg.cell^{-1})	2.2 (pg.cell^{-1})	11	360	33
		Tanaka et al 2007	Exp	*Stylophora pistillata*		312 ± 24	49 ± 5.6		7.5 ± 0.3		
		Godinot et al. 2011	Exp	*Stylophora pistillata*		343.2 ± 22.8	63 ± 9.8	1.6 ± 0.09	6.4	550.0	86.5
		McCauley & Goulet 2019	Exp	*Pseudopleaxura porosa*					11.9		
				Eunicea tourneforti					10.3		
		Tanaka 2017	Exp	*Montipora digitata*		132			7.9		
				Porites cylindrica		309			6.6		
NO3	Host	Tanaka 2017	Exp	*Montipora digitata*		360			7.2		
				Porites cylindrica		600			6.7		
	Symbiont	Tanaka 2017	Exp	*Montipora digitata*		132			5.2		
				Porites cylindrica		309			5.9		
NH_4	Coral holobiont	Cox and Ward 2002	Exp	*Montipora capitata*	20 µM	48.8 ± 11.9 (%)	5.7 ± 0.87 (%)				

	Animal tissue	McCauley & Goulet 2019	Exp	*Pseudopleaxura porosa*	10 µM				7		
				Eunicea tourneforti	10 µM				5.8		
		Muscatine et al. 1989	Exp	*Stylophora pistillata*	20 µM	766 ± 118.2	152.2 ± 3.4		5.88 ± 0.78		
		McCauley & Goulet 2019	Exp	*Pseudopleaxura porosa*	50 µM				6.7		
				Eunicea tourneforti	50 µM				5.3		
	Symbiont	McCauley & Goulet 2019	Exp	*Pseudopleaxura porosa*	10 µM				11.6		
				Eunicea tourneforti	10 µM				10.7		
		Snidvong & Kinzie 1994	Exp	*Pocillopora damicornis*	15 µM	311 (pg.cell^{-1})	70 (pg.cell^{-1})	1.3 (pg.cell^{-1})	5	618	119
		Muscatine et al. 1989	Exp	*Stylophora pistillata*	20 µM	286.2 ± 24.7	75.5 ± 5.9		4.4 ± 0.2		
		McCauley & Goulet 2019	Exp	*Pseudopleaxura porosa*	50 µM				10.4		
				Eunicea tourneforti	50 µM				10.6		
PO4	Coral holobiont	Stambler et al 1991	Exp	*Stylophora pistillata*	0.5 µM				5.8	416	71
					2 µM				5.1	375	73
	Animal tissue	Muscatine et al. 1989	Exp	*Stylophora pistillata*	2 µM	493.1 ± 141.2	86.2 ± 0.8		6.69 ± 1.86		
		Godinot et al. 2011	Exp	*Stylophora pistillata*	0.5 µM	744 ± 31.2	135.8 ± 4.2	4.6 ± 0.4	6.4	416.1	65.1
					2.5 µM	782.4 ± 42	134.4 ± 8.4	5.2 ± 0.8	6.8	388.1	57.1
		McCauley & Goulet 2019	Exp	*Pseudopleaxura porosa*	4 µM				5.8		
				Eunicea tourneforti	4 µM				5.4		
	Symbiont	Muscatine et al. 1989	Exp	*Stylophora pistillata*	2 µM	207.1 ± 39.9	33.4		6.3		
		Snidvong & Kinzie 1994	Exp	*Pocillopora damicornis*	1.2 µM	235 (pg.cell^{-1})	27 (pg.cell^{-1})	1.2 (pg.cell^{-1})	10	505	50
		Godinot et al. 2011	Exp	*Stylophora pistillata*	0.5 µM	398.4 ± 34.8	65.8 ± 15.4	2 ± 0.09	7.1	518.8	73.4
					2.5 µM	490.8 ± 79.2	78.4 ± 12.6	3.1 ± 0.16	7.3	409.0	56.0
		McCauley & Goulet 2019	Exp	*Pseudopleaxura porosa*	4 µM				13.1		
			Exp	*Eunicea tourneforti*	4 µM				9.1		
NO_3 / PO_4	Coral holobiont	Tanaka et al 2010	Exp	*Montipora digitata*	10.4 µM 0.49 µM N:P 21	3108 ± 252	420 ± 14	28.5 ± 1.2	8.6	282	33

NO_3 / PO_4	Animal tissue	Tanaka et al 2007	Exp (5 days)	*Stylophora pistillata*	5 µM	5940 ± 480	882 ± 84		7.9 ± 0.1		
			Exp (10 days)	*Stylophora pistillata*	0.3 µM N:P : 16	7056 ± 672	1008 ± 98		8.1 ± 0.1		
		Tanaka 2017	Exp	*Montipora digitata*		456			7.1		

				Porites cylindrica		657			6.6		
	Symbiont	Tanaka et al 2007	Exp (5 days)	*Stylophora pistillata*	N : 5 µM<br>P : 0.3 µM<br>N:P : 16	456 ± 36	62 ± 5.6		8.8 ± 0.3		
			Exp (10 days)			624 ± 60	84 ± 8.4		8.7 ± 0.2		
		Tanaka 2017	Exp	*Montipora digitata*	N : 1.5 µM<br>P : 0.1 µM<br>N:P 15	192			6.4		
				Porites cylindrica		402			6.1		
NH_4 / PO_4	Coral holobiont	Meyer & Shultz 1985	In situ (fish excretion)	*Acropora palmata*	N : 2.4 mmol m^{-2} d^{-1}<br>P : 0.2 mmol m^{-2} d^{-1}		440 ± 50	50 ± 5			20.7 ± 2.1
				Porites furcata	N : 7.3 mmol m^{-2} d^{-1}<br>P : 0.1 mmol m^{-2} d^{-1}		564 ± 16	48 ± 2			24.4 ± 1
	Animal tissue	Muscatine et al. 1989	Exp	*Stylophora pistillata*	N:P : 10	844.2 ± 60.2	172.4 ± 21.4		5.80 ± 1.13		
	Symbiont	Muscatine et al. 1989	Exp	*Stylophora pistillata*	N:P : 10	303.3 ± 15.8	74.6 ± 0.9		4.7 ± 0.2		
		Snidvong & Kinzie 1994	Exp	*Pocillopora damicornis*	N:P 12.5	184 (pg.$cell^{-1}$)	42 (pg.$cell^{-1}$)	0.9 (pg.$cell^{-1}$)	5	528	103

II Results

Table S3: Summary of the median and standard error (SE) for each parameter in *Turbinaria reniformis* (a.) and *Sarcophyton glaucum* (b.)

a. ***Turbinaria reniformis***

	Control balanced DIN:DIP2.5		Balanced DIN:DIP3		Imbalanced DIN:DIP0.5		Imbalanced DIN:DIP15	
	Median	± SE	Median	± SE	Median	± SE	Median	± SE
**Nitrate uptake (nmole NO3.min-1.g-1 AFDW)**	7.35	3.66	108.84	12.61	12.60	2.98	56.55	8.71
**Phosphorus uptake (nmole PO4.min-1.g-1 AFDW)**	3.17	1.41	70.39	7.77	62.79	11.41	13.19	6.48
**Total chlorophyll a and c2 (mg.g-1 AFDW)**	0.21	0.024	0.38	0.054	0.21	0.046	0.30	0.049
**Net photosynthetic rates (mmol.h-1.g-1 AFDW)**	0.06	0.007	0.14	0.016	0.09	0.018	0.09	0.007
**Respiration rates (mmol.h-1.g-1 AFDW)**	-0.13	0.01	-0.16	0.01	-0.16	0.03	-0.21	0.02
**Yield**	0.50	0.01	0.49	0.02	0.51	0.01	0.45	0.01
**Holobiont carbohydrate content (mg.g-1 AFDW)**	1.61	0.18	3.19	0.41	3.42	0.41	2.89	0.31
**Holobiont lipid content (mg.g-1 AFDW)**	9.70	0.51	18.90	3.74	8.82	2.55	14.13	1.18
**Carbon (mg.g-1 AFDW)**	24.07	2.44	39.29	5.85	47.19	2.98	35.35	1.10
**Nitrogen (mg.g-1 AFDW)**	3.77	0.60	5.95	1.00	7.78	0.41	6.40	0.23
**Phosphorous (mg.g-1 AFDW)**	0.16	0.08	0.12	0.01	0.24	0.13	0.11	0.004
**C:N (mass)**	6.39	0.67	6.60	0.13	6.15	0.19	5.52	0.10
**C:P (mass)**	161.96	49.69	325.66	11.56	218.71	57.54	318.33	18.75
**N:P (mass)**	20.36	7.90	49.31	2.72	35.61	10.16	59.35	2.74
**Dinoflagellate density (x10^6 cells.g-1 AFDW)**	425.88	42.12	258.50	25.06	135.83	46.25	317.84	19.87
**Holobiont protein content (mg.g-1 AFDW)**	94.24	3.54	102.50	3.02	79.32	8.70	100.29	4.57

b. ***Sarcophyton glaucum***

	Control balanced DIN:DIP2.5		Balanced DIN:DIP3		Imbalanced DIN:DIP0.5		Imbalanced DIN:DIP15	
	Median	± SE	Median	± SE	Median	± SE	Median	± SE
**Nitrate uptake (nmole NO3.min-1.g-1 AFDW)**	10.49	4.65	24.38	4.02	4.33	1.28	12.44	2.02
**Phosphorus uptake (nmole PO4.min-1.g-1 AFDW)**	7.67	1.94	9.52	3.76	21.24	3.01	5.95	1.11
**Total chlorophyll a and c2 (mg.g-1 AFDW)**	0.22	0.04	0.32	0.07	0.20	0.05	0.30	0.08
**Net photosynthetic rates (mmol.h-1.g-1 AFDW)**	0.02	0.002	0.01	0.005	0.02	0.002	0.01	0.003
**Respiration rates (mmol.h-1.g-1 AFDW)**	-0.03	0.003	-0.02	0.004	-0.02	0.001	-0.02	0.003
**Yield**	0.51	0.02	0.63	0.02	0.46	0.02	0.59	0.02
**Holobiont carbohydrate content (mg.g-1 AFDW)**	2.22	0.06	2.22	0.08	2.75	0.17	1.05	0.26
**Holobiont lipid content (mg.g-1 AFDW)**	446.93	50.01	474.39	36.65	389.04	46.10	355.07	76.07
**Carbon (mg.g-1 AFDW)**	210.51	5.64	191.34	28.29	194.49	23.46	218.67	5.37
**Nitrogen (mg.g-1 AFDW)**	17.14	0.45	12.08	3.92	17.06	1.79	17.68	2.15
**Phosphorous (mg.g-1 AFDW)**	0.22	0.01	0.24	0.11	0.21	0.01	0.23	0.01
**C:N (mass)**	12.15	0.09	16.16	1.79	13.09	1.91	12.15	1.40
**C:P (mass)**	918.89	36.50	848.54	228.29	973.19	122.93	964.33	52.22
**N:P (mass)**	75.78	2.45	50.80	9.51	78.46	7.30	78.43	5.26
**Dinoflagellate density (x10^6 cells.g-1 AFDW)**	314.18	16.30	294.76	10.69	292.96	18.03	361.03	24.55
**Holobiont protein content (mg.g-1 AFDW)**	36.78	2.27	39.00	8.72	69.23	10.23	26.63	6.36

Table S4: Summary of the statistical outcomes of the different measurements done on *Sarcophyton glaucum* and *Turbinaria reniformis*

a. Nutrient uptake rates

Nitrate uptake per AFDW

Sarcophyton glaucum

	Df	Sum Sq	Mean Sq	F value	P value
Condition	3	989.02	329.67	7.10	0.003
Residuals	15	696.82	46.45		

	Estimate	SE	Df	t.ratio	P value (fdr)
Control - NO_3	-0.3	4.6	15	-0.06	0.95
Control - NO_3PO_4	-13.4	4.6	15	-2.93	0.02
Control - PO_4	5.8	4.6	15	1.28	0.26
NO_3 - NO_3PO_4	-13.1	4.3	15	-3.04	0.02
NO_3PO_4 - PO_4	19.3	4.3	15	4.47	0.003
NO_3 - PO_4	6.1	4.3	15	1.42	0.26

Turbinaria reniformis

	Df	Kruskal-Wallis Chi-squared	P value
Condition	3	16.70	0.001

	Estimate	SE	Df	t.ratio	P value (fdr)
Control - NO_3	-48.1	13.8	16	-3.48	0.005
Control - NO_3PO_4	-107.3	13.8	16	-7.77	< 0.001
Control - PO_4	-4.5	15.1	16	-0.30	0.77
NO_3 - NO_3PO_4	-59.2	12.3	16	-4.80	0.00
NO_3PO_4 - PO_4	102.8	13.8	16	7.45	< 0.001
NO_3 - PO_4	43.6	13.8	16	3.16	0.01

Comparaison S. glaucum vs T.reniformis in control condition

Welch two sample t-test		
t	df	p-value
0.44	5	0.68

Phosphate uptake per AFDW

Sarcophyton glaucum

	Df	Sum Sq	Mean Sq	F value	P value
Condition	3	491.38	163.794	4.0547	0.024
Residuals	17	686.73	40.396		

	Estimate	SE	Df	t.ratio	P value (fdr)
Control - NO_3	1.6	4.1	17	0.40	0.98
Control - NO_3PO_4	-6.1	4.1	17	-1.49	0.46
Control - PO_4	-10.5	4.3	17	-2.46	0.10
NO_3 - NO_3PO_4	-7.7	3.7	17	-2.11	0.19
NO_3PO_4 - PO_4	-4.4	3.9	17	-1.14	0.67
NO_3 - PO_4	-12.1	3.9	17	-3.15	0.03

Turbinaria reniformis

	Df	Sum Sq	Mean Sq	F value	P value
Condition	3	18563.4	6187.8	15.9	< 0.001
Residuals	18	7001.7	389		

	Estimate	SE	Df	t.ratio	P value (fdr)
Control - NO_3	-11.9	12.7	18	-0.94	0.79
Control - NO_3PO_4	-71.0	12.7	18	-5.58	< 0.001
Control - PO_4	-57.0	12.7	18	-4.47	0.001
NO_3 - NO_3PO_4	-59.1	12.7	18	-5.19	< 0.001
NO_3PO_4 - PO_4	-14.1	12.7	18	1.24	0.61
NO_3 - PO_4	-45.1	12.7	18	-3.96	0.01

Comparaison S. glaucum vs T.reniformis in control condition

Welch two sample t-test		
t	df	p-value
1.45	5.48	0.20

b. Physiological parameters

Chlorophyll a and c_2 content per Ash free Dry Weight

Sarcophyton glaucum

	Df	Sum Sq	Mean Sq	F value	P value
**Condition**	3	0.19	0.06	2.90	0.06
**Residuals**	20	0.45	0.02		

Turbinaria reniformis

	Df	Sum Sq	Mean Sq	F value	P value
**Condition**	3	0.15	0.05	4.40	0.02
**Residuals**	19	0.22	0.01		

	Estimate	SE	Df	t.ratio	P value (fdr)
**Control - NO_3**	-0.1	0.1	19	-1.76	0.19
**Control - NO_3PO_4**	-0.2	0.1	19	-3.35	0.02
**Control - PO_4**	0.0	0.1	19	-0.46	0.65
**NO_3 - NO_3PO_4**	0.1	0.1	19	-1.59	0.19
**NO_3PO_4 - PO_4**	0.2	0.1	19	2.74	0.04
NO_3 - PO_4	0.1	0.1	19	1.22	0.29

Comparaison S. glaucum vs T.reniformis in control condition

Welch two sample t-test		
**t**	**df**	**p-value**
-0.37	8	0.72

Net Photosynthesis rates per AFDW

Sarcophyton glaucum

	Df	Sum Sq	Mean Sq	F value	P value
**Condition**	3	0.0001	4.6×10^{-5}	0.72	0.55
**Residuals**	20	0.001	6.4×10^{-5}		

Turbinaria reniformis

	Df	Sum Sq	Mean Sq	F value	P value
**Condition**	3	0.02	0.01	6.78	0.002
**Residuals**	20	0.02	0.001		

	Estimate	SE	Df	t.ratio	P value (fdr)
**Control - NO_3**	-0.03	0.02	20	-1.48	0.41
**Control - NO_3PO_4**	-0.08	0.02	20	-4.44	0.002
**Control - PO_4**	-0.02	0.02	20	-0.97	0.77
**NO_3 - NO_3PO_4**	-0.05	0.02	20	-2.96	0.06
**NO_3PO_4 - PO_4**	0.06	0.02	20	3.47	0.02
NO_3 - PO_4	0.01	0.02	20	0.50	0.93

Comparaison S. glaucum vs T.reniformis in control condition

Welch two sample t-test		
**t**	**df**	**p-value**
-5.04	5	0.002

Respiration rates per ash free dry weight

Sarcophyton glaucum

	Df	Sum Sq	Mean Sq	F value	P value
**Condition**	3	0.0002	6.16×10^{-5}	1.22	0.33
**Residuals**	20	0.0010	5.06×10^{-5}		

Turbinaria reniformis

	Df	Kruskla-Wallis Chi-squared	P value
**Condition**	3	6.33	0.10

Comparaison S. glaucum vs T.reniformis in control condition

Wilcoxon rank sum test	
**W**	**p-value**
0	0.002

Photosystem efficiency Yield (Fv/Fm)

Sarcophyton glaucum

	Df	Sum Sq	Mean Sq	F value	P value
**Condition**	3	0.09	0.03	9.10	0.0002
**Residuals**	28	0.09	0.003		

	Estimate	SE	Df	t.ratio	P value (fdr)
**Control - NO_3**	-0.06	0.03	28	-2.02	0.20
**Control - NO_3PO_4**	-0.10	0.03	28	-3.49	0.01
**Control - PO_4**	0.04	0.03	28	1.33	0.56
**NO_3 - NO_3PO_4**	-0.04	0.03	28	-1.47	0.47
**NO_3PO_4 - PO_4**	0.14	0.03	28	4.82	< 0.001
NO_3 - PO_4	0.10	0.03	28	3.35	0.01

Turbinaria reniformis

	Df	Sum Sq	Mean Sq	F value	P value
**Condition**	3	0.021	0.007	6.612	0.002
**Residuals**	28	0.029	0.001		

	Estimate	SE	Df	t.ratio	P value (fdr)
**Control - NO_3**	0.05	0.02	28	3.33	0.01
**Control - NO_3PO_4**	0.01	0.02	28	0.43	0.97
**Control - PO_4**	-0.01	0.02	28	-0.87	0.82
**NO_3 - NO_3PO_4**	-0.05	0.02	28	-2.90	0.03
**NO_3PO_4 - PO_4**	-0.02	0.02	28	-1.29	0.58
NO_3 - PO_4	-0.07	0.02	28	-4.19	0.00

Comparaison S. glaucum vs T.reniformis in control condition

Wilcoxon rank sum test	
**W**	**p-value**
44	0.23

Carbohydrate per Ash free dry weight

Sarcophyton glaucum

	Df	Sum Sq	Mean Sq	F value	P value
**Condition**	3	9.49	3.16	19.19	< 0.0001
**Residuals**	19	3.13	0.16		

	Estimate	SE	Df	t.ratio	P value (fdr)
**Control - NO_3**	1.05	0.25	19	4.29	0.008
**Control - NO_3PO_4**	-0.07	0.25	19	-0.26	0.79
**Control - PO_4**	-0.70	0.25	19	-2.84	0.02
**NO_3 - NO_3PO_4**	-1.12	0.23	19	-4.77	0.004
**NO_3PO_4 - PO_4**	-0.63	0.23	19	-2.71	0.02
NO_3 - PO_4	-1.75	0.23	19	-7.48	< 0.001

Turbinaria reniformis

	Df	Sum Sq	Mean Sq	F value	P value
**Condition**	3	11.45	3.82	5.92	0.00
**Residuals**	19	12.25	0.64		

	Estimate	SE	Df	t.ratio	P value (fdr)
**Control - NO_3**	-1.05	0.46	19	-2.27	0.14
**Control - NO_3PO_4**	-1.66	0.49	19	-3.41	0.01
**Control - PO_4**	-1.76	0.46	19	-3.80	0.01
**NO_3 - NO_3PO_4**	-0.60	0.49	19	-1.24	0.61
**NO_3PO_4 - PO_4**	-0.10	0.49	19	-0.21	1.00
NO_3 - PO_4	-0.71	0.46	19	-1.53	0.44

Comparaison S. glaucum vs T.reniformis in control condition

Welch two sample t-test		
**t**	**df**	**p-value**
2.29	6	0.06

Lipid per Ash free dry weight

Sarcophyton glaucum

	Df	Sum Sq	Mean Sq	F value	P value
**Condition**	3	47296	15765	0.89	0.46
**Residuals**	20	352694	17635		

Turbinaria reniformis

	Df	Kruskal-Wallis Chi-squared	P value
**Condition**	3	11.1	0.02

	Estimate	SE	Df	t.ratio	P value (fdr)
**Control - NO_3**	-4.88	3.33	20	-1.47	0.22
**Control - NO_3PO_4**	-9.90	3.33	20	-2.98	0.03
**Control - PO_4**	-0.31	3.33	20	-0.09	0.93
**NO_3 - NO_3PO_4**	-5.02	3.33	20	-1.51	0.22
**NO_3PO_4 - PO_4**	9.59	3.33	20	2.88	0.03
NO_3 - PO_4	4.56	3.33	20	1.37	0.22

Comparaison S. glaucum vs T.reniformis in control condition

Welch two sample t-test		
**t**	**df**	**p-value**
7.77	5	0.001

b. Elemental composition and stoichiometry

Carbon content per ash free dry weight

Sarcophyton glaucum

	Df	Kruskal-Wallis Chi-squared	P value
**Condition**	3	2.27	0.52

Turbinaria reniformis

	Df	Kruskal-Wallis Chi-squared	P value
**Condition**	3	10.70	0.01

	Estimate	SE	Df	t.ratio	P value (fdr)
**Control - NO_3**	-10.79	5.013	12	-2.15	0.19
**Control - NO_3PO_4**	-15.12	5.013	12	-3.02	0.05
**Control - PO_4**	-22.72	5.013	12	-4.53	0.003
**NO_3 - NO_3PO_4**	-4.329	5.013	12	-0.86	0.82
**NO_3PO_4 - PO_4**	-7.599	5.013	12	-1.52	0.46
**NO_3 - PO_4**	-11.927	5.013	12	-2.38	0.13

Comparaison S. glaucum vs T.reniformis in control condition

Wilcoxon rank sum test	
**W**	**p-value**
16	0.03

Nitrogen content per ash free dry weight

Sarcophyton glaucum

	Df	Kruskal-Wallis Chi-squared	P value
**Condition**	3	1.74	0.63

Turbinaria reniformis

	Df	Kruskal-Wallis Chi-squared	P value
**Condition**	3	8.63	0.03

	Estimate	SE	Df	t.ratio	P value (fdr)
**Control - NO_3**	-2.72	0.89	12	-3.06	0.03
**Control - NO_3PO_4**	-2.36	0.89	12	-2.65	0.04
**Control - PO_4**	-3.93	0.89	12	-4.41	0.01
**NO_3 - NO_3PO_4**	0.36	0.89	12	0.41	0.69
**NO_3PO_4 - PO_4**	-1.57	0.89	12	-1.76	0.16
**NO_3 - PO_4**	-1.21	0.89	12	-1.35	0.24

Comparaison S. glaucum vs T.reniformis in control condition

Wilcoxon rank sum test	
**W**	**p-value**
16	0.03

Carbon : Nitrogen ratio per ash free dry weight

Sarcophyton glaucum

	Df	Kruskal-Wallis Chi-squared	P value
**Condition**	3	1.57	0.67

Turbinaria reniformis

	Df	Kruskal-Wallis Chi-squared	P value
**Condition**	3	9.55	0.02

	Estimate	SE	Df	t.ratio	P value (fdr)
**Control - NO_3**	1.46	0.51	12	2.88	0.08
**Control - NO_3PO_4**	0.34	0.51	12	0.67	0.52
**Control - PO_4**	0.76	0.51	12	1.50	0.29
**NO_3 - NO_3PO_4**	-1.12	0.51	12	-2.21	0.14
**NO_3PO_4 - PO_4**	0.42	0.51	12	0.83	0.51
**NO_3 - PO_4**	-0.70	0.51	12	-1.37	0.29

Comparaison S. glaucum vs T.reniformis in control condition

Wilcoxon rank sum test	
**W**	**p-value**
16	0.03

Phosphorus content

Sarcophyton glaucum

	Df	Kruskal-Wallis Chi-squared	P value
Condition	3	0.71	0.87

Turbinaria reniformis

	Df	Kruskal-Wallis Chi-squared	P value
Condition	3	5.38	0.15

Comparaison S. glaucum vs T.reniformis in control condition

Welch two sample t-test		
t	df	p-value
0.21	3	0.85

Carbon : Phosphorus ratio per ash free dry weight

Sarcophyton glaucum

	Df	Sum Sq	Mean Sq	F value	P value
Condition	3	15704	5235	0.07	0.97
Residuals	12	855414	71284		

Turbinaria reniformis

	Df	Kruskal-Wallis Chi-squared	P value
Condition	3	9.73	0.02

	Estimate	SE	Df	t.ratio	P value (fdr)
Control - NO_3	-163.37	55.97	12	-2.92	0.05
Control - NO_3PO_4	-158.60	55.97	12	-2.83	0.06
Control - PO_4	-42.74	55.97	12	-0.76	0.87
NO_3 - NO_3PO_4	4.77	55.97	12	0.09	1.00
NO_3PO_4 - PO_4	115.86	55.97	12	2.07	0.22
NO_3 - PO_4	120.63	55.97	12	2.16	0.19

Comparaison S. glaucum vs T.reniformis in control condition

Wilcoxon rank sum test	
W	p-value
16	0.029

Nitrogen : Phosphorus ratio per ash free dry weight

Sarcophyton glaucum

	Df	Kruskal-Wallis Chi-squared	P value
Condition	3	3.0221	0.3882

Turbinaria reniformis

	Df	Kruskal-Wallis Chi-squared	P value
Condition	3	9.7941	0.02

	Estimate	SE	Df	t.ratio	P value (fdr)
Control - NO_3	-35.74	9.5	12	-3.76	0.01
Control - NO_3PO_4	-25.15	9.5	12	-2.65	0.09
Control - PO_4	-10.46	9.5	12	-1.10	0.70
NO_3 - NO_3PO_4	10.59	9.5	12	1.12	0.69
NO_3PO_4 - PO_4	14.68	9.5	12	1.55	0.44
NO_3 - PO_4	25.28	9.5	12	2.66	0.08

Comparaison S. glaucum vs T.reniformis in control condition

Welch two sample t-test		
t	df	p-value
6.48	3	0.004

c. Dinoflagellate density and protein content

Dinoflagellate density

Supplementary

Sarcophyton glaucum

	Df	Sum Sq	Mean Sq	F value	P value
**Condition**	3	10087	3362.3	1.71	0.20
**Residuals**	20	39234	1961.7		

Turbinaria reniformis

	Df	Sum Sq	Mean Sq	F value	P value
**Condition**	3	0.15	55194	7.46	0.002
**Residuals**	19	0.22	7403		

	Estimate	SE	Df	t.ratio	P value (fdr)
**Control - NO_3**	53.61	49.68	20	1.08	0.29
**Control - NO_3PO_4**	108.84	49.68	20	2.19	0.06
**Control - PO_4**	224.14	49.68	20	4.51	0.001
**NO_3 - NO_3PO_4**	55.23	49.68	20	1.11	0.29
**NO_3PO_4 - PO_4**	115.3	49.68	20	2.32	0.06
**NO_3 - PO_4**	170.53	49.68	20	3.43	0.01

Comparaison S. glaucum vs T.reniformis in control condition

Welch two sample t-test		
**t**	**df**	**p-value**
-1.66	6	0.14

Protein holobiont content

Sarcophyton glaucum

	Df	Sum Sq	Mean Sq	F value	P value
**Condition**	3	5726.5	1908.83	5.6226	0.005809
**Residuals**	20	6789.9	339.49		

	Estimate	SE	Df	t.ratio	P value (fdr)
**Control - NO_3**	6.99	10.6	20	0.66	0.52
**Control - NO_3PO_4**	-9.94	10.6	20	-0.93	0.43
**Control - PO_4**	-33.84	10.6	20	-3.18	0.01
**NO_3 - NO_3PO_4**	-16.93	10.6	20	-1.59	0.19
**NO_3PO_4 - PO_4**	-23.90	10.6	20	-2.25	0.07
**NO_3 - PO_4**	-40.23	10.6	20	-3.84	0.01

Turbinaria reniformis

	Df	Sum Sq	Mean Sq	F value	P value
**Condition**	3	1952.9	650.97	4.43	0.02
**Residuals**	19	2789.3	146.81		

	Estimate	SE	Df	t.ratio	P value (fdr)
**Control - NO_3**	-8.7	7	19	-1.24	0.34
**Control - NO_3PO_4**	-6.56	7	19	-0.94	0.43
**Control - PO_4**	15.8	7.34	19	2.15	0.09
**NO_3 - NO_3PO_4**	2.14	7	19	0.31	0.76
**NO_3PO_4 - PO_4**	22.36	7.34	19	3.05	0.02
**NO_3 - PO_4**	24.5	7.34	19	3.34	0.02

Comparaison S. glaucum vs T.reniformis in control condition

Wilcoxon rank sum test	
**W**	**p-value**
0	0.002

III Materials and Methods

Table S5. Example of nubbin repartition for the different measurements in one condition and one coral species

We used 6 nubbins per condition/species, with three nubbins from each aquarium
Measurement 1: Nutrient uptake rate, Photosynthesis rates, PAM, chlorophyll content, dinoflagellate density and protein content
Measurement 2: Lipid and carbohydrate content
Measurement 3: Lipid peroxidation (LPO) and non-enzymatic total antioxidant capacity (NETAC)
Measurement 4: Carbon, nitrogen and phosphorus content

	Nubbin number (each from a different colony)											
	1	2	3	4	5	6	7	8	9	10	11	12
Aquarium 1	1	3	4	2	3	1		4	2	1	3	2
Aquarium 2	2	4	1	3		3	2	1	4	3	2	1

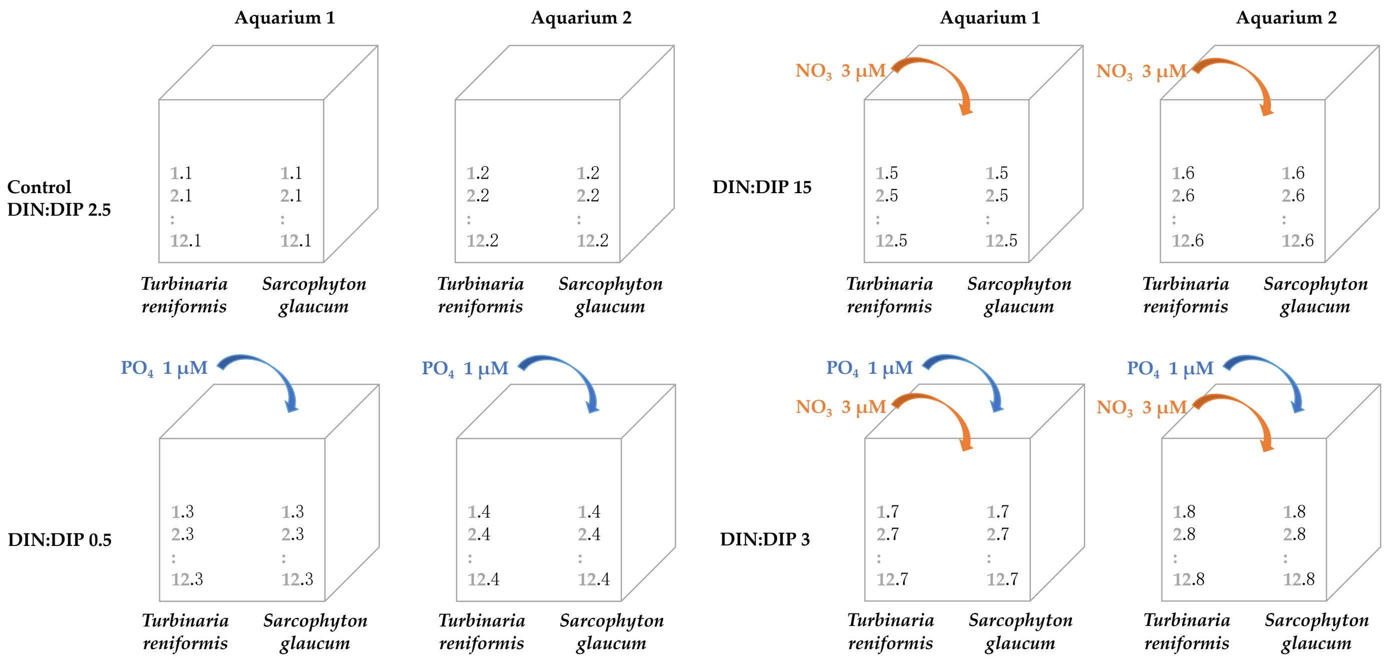

Figure S1. Summary of the experimental design and repartition of nubbins across treatment and aquaria.

Each aquarium has 24 nubbins in total, with12 nubbins per species (*Turbinaria reniformis* and *Sarcophyton glaucum*). The **numbers from 1 to 12 in grey** represent the ID number of the mother colony. The **numbers from 1 to 8 in black** represent the id number of the nubbin cut from the mother colony. So in each aquaria there is one nubbin from each colony
